# Intra-tumoral angiogenesis correlates with immune features and prognosis in glioma

**DOI:** 10.18632/aging.204079

**Published:** 2022-05-17

**Authors:** Qing Zhang, Yao-Xing Guo, Wan-Lin Zhang, Hai-Yan Lian, Natasha Iranzad, Endi Wang, Ying-Chun Li, Hai-Chao Tong, Le-Yao Li, Ling-Yun Dong, Lian-He Yang, Shuang Ma

**Affiliations:** 1 Department of Pathology, The First Affiliated Hospital and College of Basic Medical Sciences, China Medical University, Shenyang, Liaoning, China; 2 Department of Neurology, Sheng Jing Hospital of China Medical University, Shenyang, Liaoning, China; 3 Department of Pathology, Hebei Petro China Central Hospital, Langfang, Hebei, China; 4 Department of Ophthalmology, Jili Hospital of Liuyang (Liuyang Eye Hospital), Changsha, Hunan, China; 5 Department of Pathology, Duke University Medical Center, Durham, NC 27710, USA

**Keywords:** glioma, angiogenesis, malignant phenotype, prognosis, immune characteristics, therapy sensitivity

## Abstract

Gliomas are the most common malignant tumor in the brain. As with other tumors, the progression of glioma depends on intra-tumoral angiogenesis. However, the effect of angiogenesis on gliomas is still not fully understood. In this study, we developed an angiogenesis pathway score using Gene Set Variation Analysis (GSAV) in R to assess the status of intra-glioma angiogenesis in The Cancer Genome Atlas (TCGA), Chinese Glioma Genome Atlas (CGGA mRNAseq_325, CGGA mRNA-array), and GSE16011 datasets. We found that the angiogenesis pathway score not only accurately predicted the prognosis of glioma patients, but also accurately distinguished the malignant phenotype and immune characteristics of gliomas. In addition, as an independent prognostic factor, the score could predict glioma sensitivity to radiotherapy and chemotherapy. In summary, we used the angiogenesis pathway score to reveal the relationship between glioma angiogenesis and the malignant phenotype, immune characteristics, and prognosis of glioma.

## INTRODUCTION

As the most frequent malignant primary intracranial tumor, gliomas account for approximately 80% of brain tumors and can be further divided into glioblastoma (GBM) and low-grade gliomas (LGG) based on histology and molecular features [1]. Although many advancements in surgery-related technologies and neoadjuvant chemotherapy have been developed in recent years, the prognosis of glioma patients has not been significantly improved [2]. This is especially true for GBM patients, who have a median survival time of 15 months [3]. Hence, the need to find more effective treatment methods to improve the prognosis of glioma patients is great.

The pathophysiologic mechanisms underlying angiogenesis have attracted much attention in research due to its vital role in wound repair and cancer pathogenesis. In cancer, the outgrowth of capillary sprouts is more prominent and continuous than that in inflammation or in fresh wounds, which indicates that tumor progression partially relies on angiogenesis [4]. Compared to tissue blood vessels, tumor vasculature is less mature, much more tortuous, dilated, and disorganized, and demonstrates a lack of mural cell association. Moreover, vascular heterogeneity, with both hyper- and hypo-vascular regions seen within the same tumor, is also a typical feature of tumoral blood vessels [5].

Tumor angiogenesis is regulated by many factors including vascular endothelial growth factors (VEGFs) and their receptors (VEGFRs), fibroblast growth factors (FGFs) and their receptors, angiopoietin (ANGPT), tyrosine kinase with immunoglobulin-like and EGF-like domains (TIE) signaling, and also transforming growth factor-betas (TGF-βs). They play a vital role in angiogenesis in various types of tumors [6]. In addition, various tumor-associated stromal cells and the extracellular matrix in which they are embedded also play an important role in the maintenance of angiogenesis during tumor progression [7]. Tumor-associated macrophages are a prime example. They secrete inflammatory cytokines and growth factors to support angiogenesis by promoting endothelial cell survival, activation, and proliferation [8–10]. Although research exploring the mechanism of tumor angiogenesis has been performed, its pathogenesis has yet to be fully elucidated. Thus, further investigation is necessary to advance our understanding of this process.

Angiogenesis affects tumor cells and their microenvironment. Blood vessels provide tumor cells with oxygen and nutrients. They also supply a route for tumor cells to metastasize which promotes the malignant phenotype of tumors [11]. In breast [12], prostate [13], lung [14], and ovarian cancers [15], high microvessel density has been identified as an independent unfavorable prognostic factor. In addition, tumor endothelial cells can promote tumor progression by down-regulating the expression of tumor suppressor factors, such as Slit2 [16]. Furthermore, tumor angiogenesis can also affect anti-tumor immunity. *In vivo* and *in vitro* experiments indicate that adhesion of leukocytes to tumor vascular endothelial cells is significantly reduced, which in turn leads to changes in the immune cell composition within the tumor microenvironment [17, 18].

Angiogenesis also has an important influence on glioma malignant phenotype, including cancer cell invasion, stem cell phenotype, genetic instability, altered metabolism, epithelial to mesenchymal transition (EMT), and anti-tumor immunosuppression [19–21]. It is precisely because of the important role of angiogenesis in tumor progression that anti-angiogenesis has become an important method for the treatment of tumors, including glioma [22]. However, the interaction between tumors and angiogenesis appears far more complicated than initially supposed [23]. For instance, there is increasing evidence that cancer cells can circumvent anti-angiogenic therapies and develop resistance to targeted monotherapy [24, 25], which is also common in gliomas [26]. Therefore, a comprehensive understanding of the underlying specific mechanisms of angiogenesis will help us discover novel and effective therapeutic targets in the anti-angiogenesis treatment of gliomas.

In this study, we used Gene Set Variation Analysis (GSVA) score to define the “HALLMARKER_ANGIOGENESIS” gene set score. The angiogenesis pathway score was associated with the state of angiogenesis in gliomas and also accurately distinguished the malignant phenotypes and immune characteristics of gliomas. In addition, the angiogenesis pathway score independently predicted prognosis and glioma sensitivity to treatment.

## RESULTS

### High angiogenesis pathway score is associated with high expression of angiogenesis- and hypoxia-related markers

**Figure 1:**
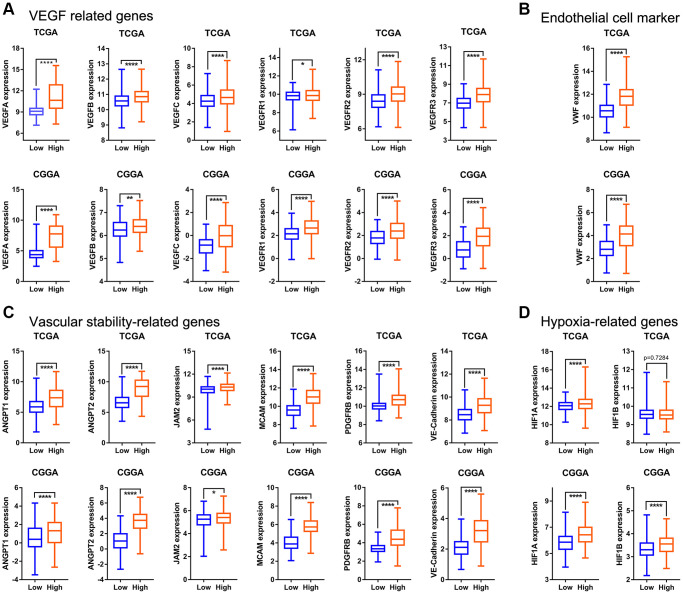
**The relationship between angiogenesis pathway score and angiogenesis regulatory factors in the TCGA and CGGA datasets.** (**A**) Expression of VEGF-related genes (VEGFA, VEGFB, VEGFC, VEGFR1, VEGFR2, VEGFR3) in high- and low-score groups. (**B**) VWF (endothelial cell marker) expression in different groups. (**C**) Expression levels of ANGPT1, ANGPT2, JAM2, MCAM, PDGFRB, and VE-Cadherin in different groups. (**D**) Gene expression levels of hypoxia-related genes (HIF1A and HIF1B).

The GSVA score of the “HALLMARK_ANGIOGENESIS” gene set retrieved from MSigDb was determined by using the global glioma gene expression data and used as angiogenesis pathway score. The median value of the angiogenesis pathway score divided a given cohort into high- and low-score groups. To explore whether the angiogenesis pathway score could reflect the level of intra-tumoral angiogenesis, we compared the expression of angiogenesis-related genes in the high- and low-score groups in the TCGA, CGGA, and CGGA (array) datasets. As shown in Figure 1A and Supplementary Figure 1A, VEGFs, including VEGFA, VEGFB, VEGFC, VEGFR1, VEGFR2, and VEGFR3, were significantly upregulated in the high-score group. This indicates that the angiogenesis pathway score can adequately reflect intra-tumoral angiogenesis. To further confirm this conclusion, we compared the expression levels of endothelial cell surface markers (CD31 and VWF) in the high- and low-score groups in the TCGA, CGGA, CGGA (array), and GSE16011 datasets (there is no expression information of CD31 in the CGGA database). Expression levels of CD31 and VWF were notably higher in the high-score group than in the low-score group (Figure 1B, Supplementary Figure 1B and 1E). This further supports the notion that a high angiogenesis pathway score is associated with a higher concentration of blood vessels within the glioma microenvironment.

In contrast to vessels in nonneoplastic tissue, tumor vasculature is highly chaotic often showing dilation and tortuous growth patterns [27]. This may be attributable to tumor vessels staying in a continuous dynamic state of growth, regression, and remodeling [28]. In addition, there is often heterogeneity in the distribution of blood vessels within the tumor microenvironment [5]. Based on these features, we infer that there is a contradiction between angiogenesis and anti-angiogenesis in the tumor. Vascular stability refers to the properties of blood vessels in a stable state. The current view is that stable blood vessels should at least have complete peritubular extracellular matrix (especially vascular basement membrane) and perivascular cell aggregation [28]. The stable state of blood vessels is antithetical to vascular remodeling and angiogenesis. In order to explore whether this contradiction exists in gliomas, we compared the expression levels of vascular stability-related markers (ANGPT1, ANGPT2, JAM2, MCAM, PDGFRB, and VE-Cadherin) in the high- and low-score glioma groups in the TCGA, CGGA, and CGGA (array) datasets. Expression levels of all the markers in the high-score group were significantly higher than those in the low-score group (Figure 1C, Supplementary Figure 1C). This indicates that angiogenesis and the maintenance of blood vessel stability exist simultaneously in gliomas, which highlights the heterogeneity of blood vessels within the glioma microenvironment.

In addition to being regulated by glioma cells, angiogenesis is also regulated by the environment. Hypoxia is an important environmental factor that regulates angiogenesis [29]. Thus, we compared the expression level of hypoxia-inducible factors in the high- and low-score groups and found that the expression level of HIF1A in the high-score group was also significantly higher than that in the low-score group in the TCGA, CGGA, and CGGA (array) datasets (Figure 1D, Supplementary Figure 1D). HIF1B was upregulated in high-score glioma group in the CGGA dataset, but not in the TCGA cohort (Figure 1D). This confirms that angiogenesis in gliomas is regulated, at least partially, by hypoxia.

### The angiogenesis pathway score demonstrated a strong ability to predict prognosis in glioma patients

**Figure 2:**
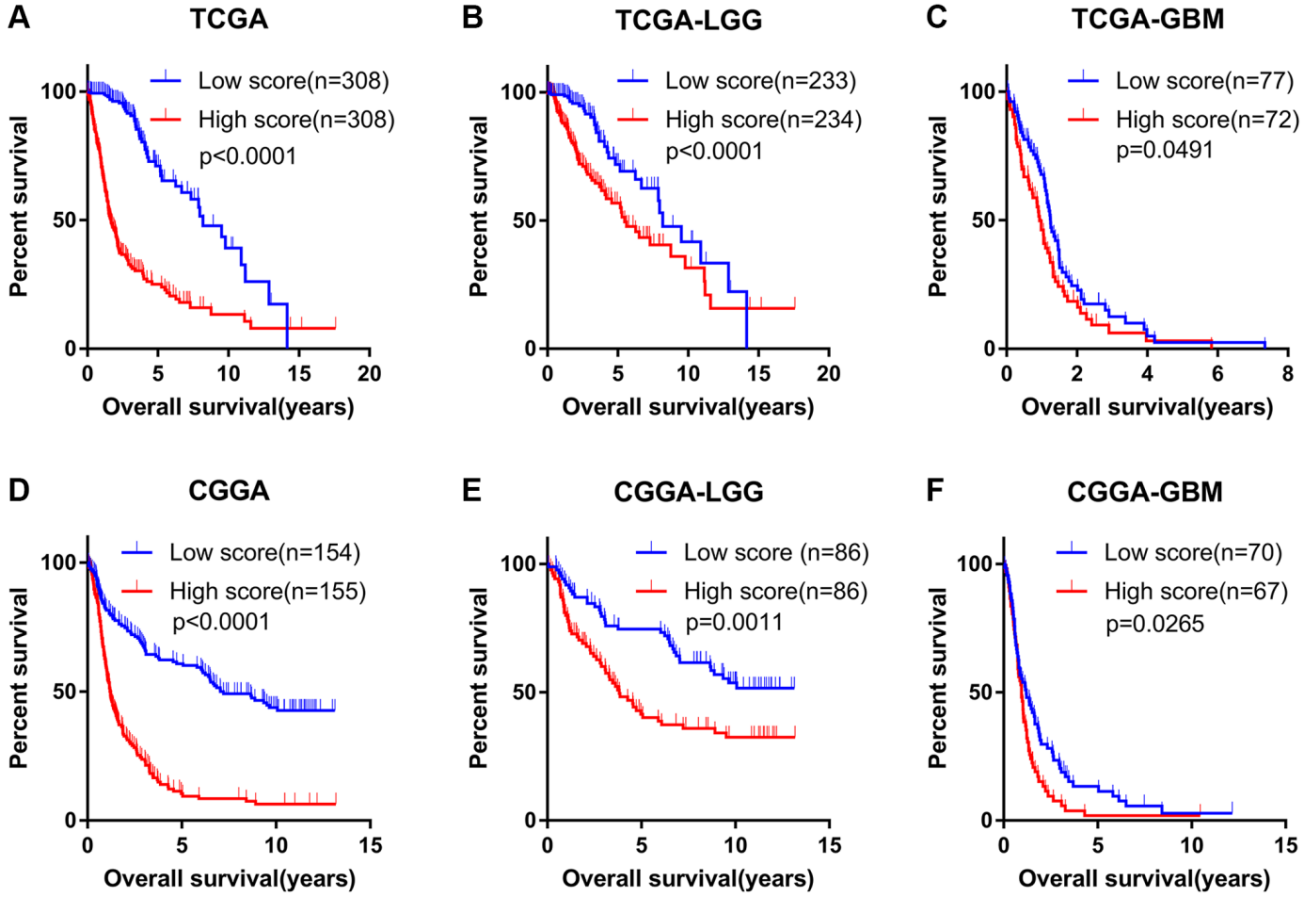
**Angiogenesis score accurately predicted the prognosis of glioma patients.** (**A**–**C**) The OS of high-score glioma (**A**), low grade glioma (LGG) (**B**) and glioblastoma (GBM) (**C**) patients was significantly shorter than that of the low-score group in the TCGA cohorts. (**D**–**F**) The OS of high-score glioma (**D**), low grade glioma (LGG) (**E**) and glioblastoma (GBM) (**F**) patients was significantly shorter than that of the low-score group in the CGGA cohorts.

In TCGA dataset, KM analysis showed that glioma patients in the low-score group displayed significantly longer OS than that seen in the high-score group (Figure 2A). In addition, following stratification by World Health Organization (WHO) grade, the prognostic value of the angiogenesis pathway score was consistent with that seen overall in the TCGA cohort (Figure 2B, 2C). Furthermore, KM analyses conducted in the CGGA, CGGA (array) and GSE16011 datasets evaluating the prognostic value of the angiogenesis pathway score obtained consensus results (Figure 2D–2F, Supplementary Figure 2A–2F). These results demonstrated the powerful potential of the angiogenesis pathway score in predicting prognosis in glioma patients.

### High angiogenesis pathway score exhibited a predilection for malignant subtypes of glioma

**Figure 3:**
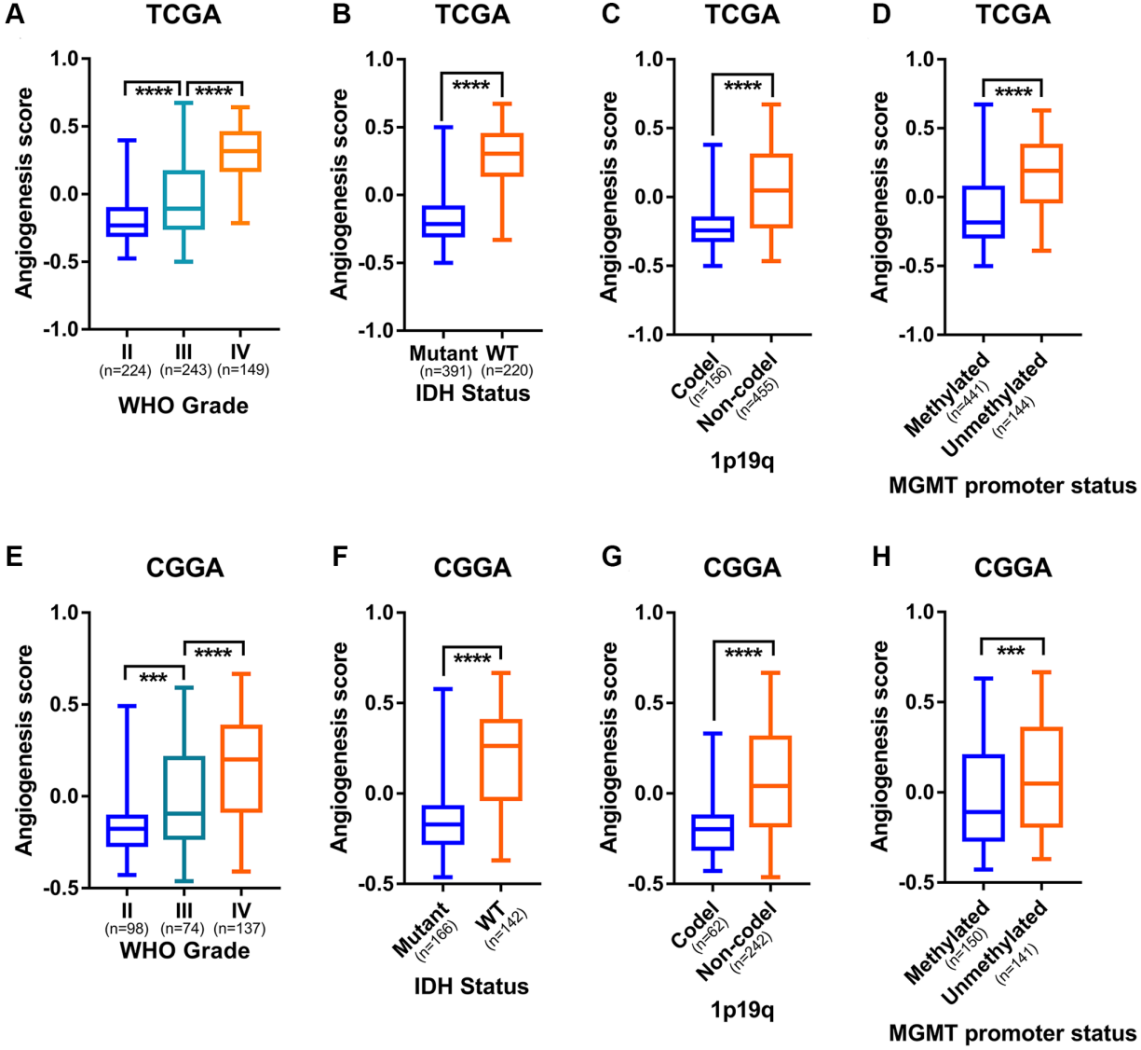
**Angiogenesis score distinguished the malignant subtypes of glioma.** (**A**) The angiogenesis score was significantly correlated with WHO grade of glioma in the TCGA cohorts. (**B**) Higher angiogenesis score enriched in IDH-wildtype gliomas than in IDH-mutant gliomas in the TCGA cohorts. (**C**) Higher angiogenesis score in 1p19q non-codeleted gliomas than in 1p19q codeleted gliomas in the TCGA cohorts. (**D**) MGMT promoter unmethylated gliomas had a significantly higher angiogenesis score than MGMT promoter methylated gliomas in the TCGA cohorts. (**E**) The angiogenesis score was significantly correlated with WHO grade of glioma in the CGGA cohorts. (**F**) Higher angiogenesis score enriched in IDH-wildtype gliomas than in IDH-mutant gliomas in the CGGA cohorts. (**G**) Higher angiogenesis score in 1p19q non-codeleted gliomas than in 1p19q codeleted gliomas in the CGGA cohorts. (**H**) MGMT promoter unmethylated gliomas had a significantly higher angiogenesis score than MGMT promoter methylated gliomas in the CGGA cohorts.

In order to explore the relationship between the angiogenesis pathway score and the clinicopathological subtypes of glioma, we examined angiogenesis pathway score with respect to WHO grade classification of glioma. In TCGA, CGGA, and CGGA (array) datasets, as the grade of glioma increased, the angiogenesis pathway score increased significantly (Figure 3A and 3E, Supplementary Figure 3A). In addition to WHO grade, it is acknowledged that IDH mutation, chromosome 1p19q codeletion, MGMT promoter methylation status, and transcriptome subtypes play a vital role in glioma progression [30, 31]. In the TCGA, CGGA, and CGGA (array) datasets, high angiogenesis pathway scores were enriched in the IDH wildtype, 1p19q non-codeleted, and MGMT promoter unmethylated gliomas (Figure 3B–3D, and 3F–3H; Supplementary Figure 3B–3D). Moreover, the mesenchymal subtype of glioma had a higher angiogenesis score than other subtypes in the TCGA and CGGA (array) datasets (Supplementary Figure 3E–3F). We collected eight samples of glioma, including cases of WHO grade II-IV, and analyzed them by bulk RNA sequencing. To further characterize the relationship between angiogenesis pathway score and clinicopathological subtypes of glioma, we scored the sequencing result by GSVA algorithm and evaluated the association of the angiogenesis pathway score with the WHO grade classification. The results showed that the WHO grade IV patients had significantly higher angiogenesis pathway scores than the WHO grade II and III patients (Supplementary Figure 3G). We also found that patients with wild type *IDH* had a significantly higher angiogenesis pathway score than those with mutant *IDH* (Supplementary Figure 3H). These are consistent with the results of previous analyses. These results indicate a close correlation between angiogenesis and aggressive pathologic features in glioma.

### The angiogenesis pathway score reflected the characteristics of glioma immune microenvironment

**Figure 4:**
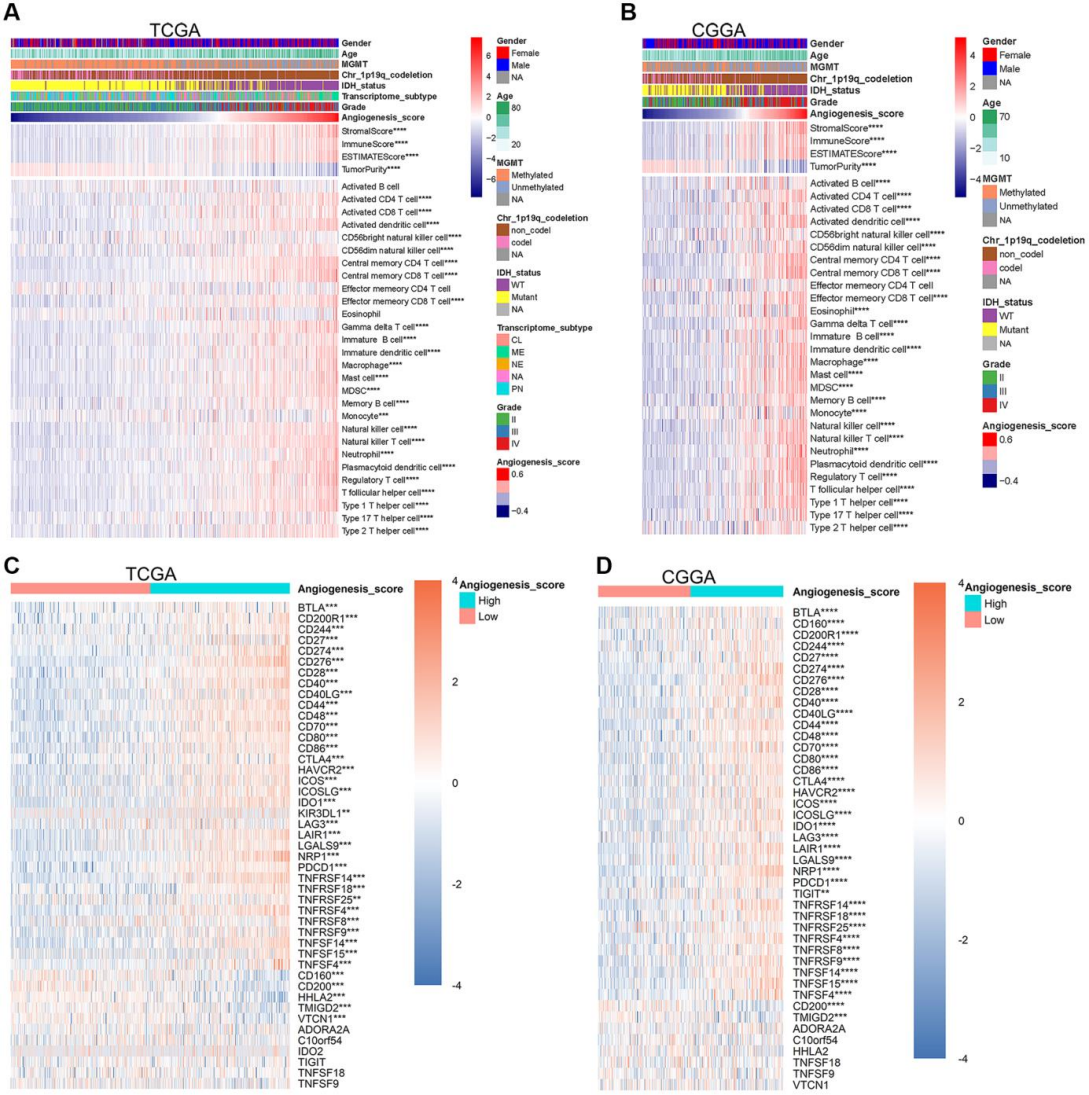
**The angiogenesis score reflected the immune characteristics of glioma.** (**A**, **B**) In the TCGA (**A**) and CGGA (**B**) datasets, with the increase of the angiogenesis pathway score, the purity of glioma was significantly reduced, the immune score was significantly increased, and the enrichment degree of most immune cells was also significantly increased. (**C**, **D**) The expression level of most immune checkpoints increased significantly as the angiogenesis score increased in the TCGA (**C**) and CGGA (**D**) cohorts.

Microvessels are an important component of the glioma microenvironment and increased angiogenesis in gliomas improves microcirculation. Bearing this in mind, we speculated the angiogenesis pathway score might be associated with the purity of glioma and the enrichment degree of immune cells within the glioma microenvironment. To examine this, we first checked correlation between the angiogenesis pathway score and glioma purity. In the TCGA, CGGA, and CGGA (array) datasets, the stromal, immune, and ESTIMATE scores were significantly positively correlated with the angiogenesis pathway score. However, the correlation between tumor purity and the angiogenesis pathway score was just the opposite (Figure 4A, 4B, Supplementary Figure 4A). Additionally, we also found that the enrichment levels of most immune cells in the high-score group were significantly higher than those in the low-score group (Figure 4A, 4B, Supplementary Figure 4A). These findings indicate that in addition to angiogenesis itself reducing the purity of gliomas, angiogenesis can also reduce glioma purity by increasing the enrichment of immune cells within the glioma microenvironment. Previous study confirmed that low glioma purity is associated with unfavorable prognosis [32]. This further conforms the accuracy of the angiogenesis pathway score in predicting the prognosis of glioma.

Many immune cells, such as CD8^+^ T cells, play an important role in the immune clearance of tumor cells, however according to our data, the high enrichment of immune cells was related to a poorer prognosis. We speculated that angiogenesis may be related to immune tolerance of gliomas. Immune checkpoints play a vital role in the maintenance of self-tolerance under normal physiological conditions and is dysregulated by tumors as a crucial factor in immune resistance [33]. Thus, we further compared the expression levels of immune checkpoints in the high- and low-score glioma groups. In the TCGA, CGGA, and CGGA (array) datasets, the expression levels of most immune checkpoints in high-score gliomas were significantly higher than those in low-score groups (Figure 4C, 4D, Supplementary Figure 4B). Our sequencing results further supported such a conclusion (Supplementary Figure 4I). Based on this, we concluded that the angiogenesis pathway score can accurately predict the status of anti-glioma immune response. PD-1 (PDCD1) and PD-L1 (CD274) are typical immune checkpoints that monoclonal antibodies (mAbs) blocking these pathways have shown significant effects in the treatment of some tumors [34–36]. The expression of PD-1 and PD-L1 were significantly positively correlated with the angiogenesis pathway score in the TCGA, CGGA, and CGGA (array) datasets (Supplementary Figure 4C–4H). All these results indicate that the higher the angiogenesis pathway score, the lower the purity of glioma and activity of the anti-glioma immune response.

### The angiogenesis pathway score correlated with regulation of inflammation in glioma and glioma metastasis

**Figure 5:**
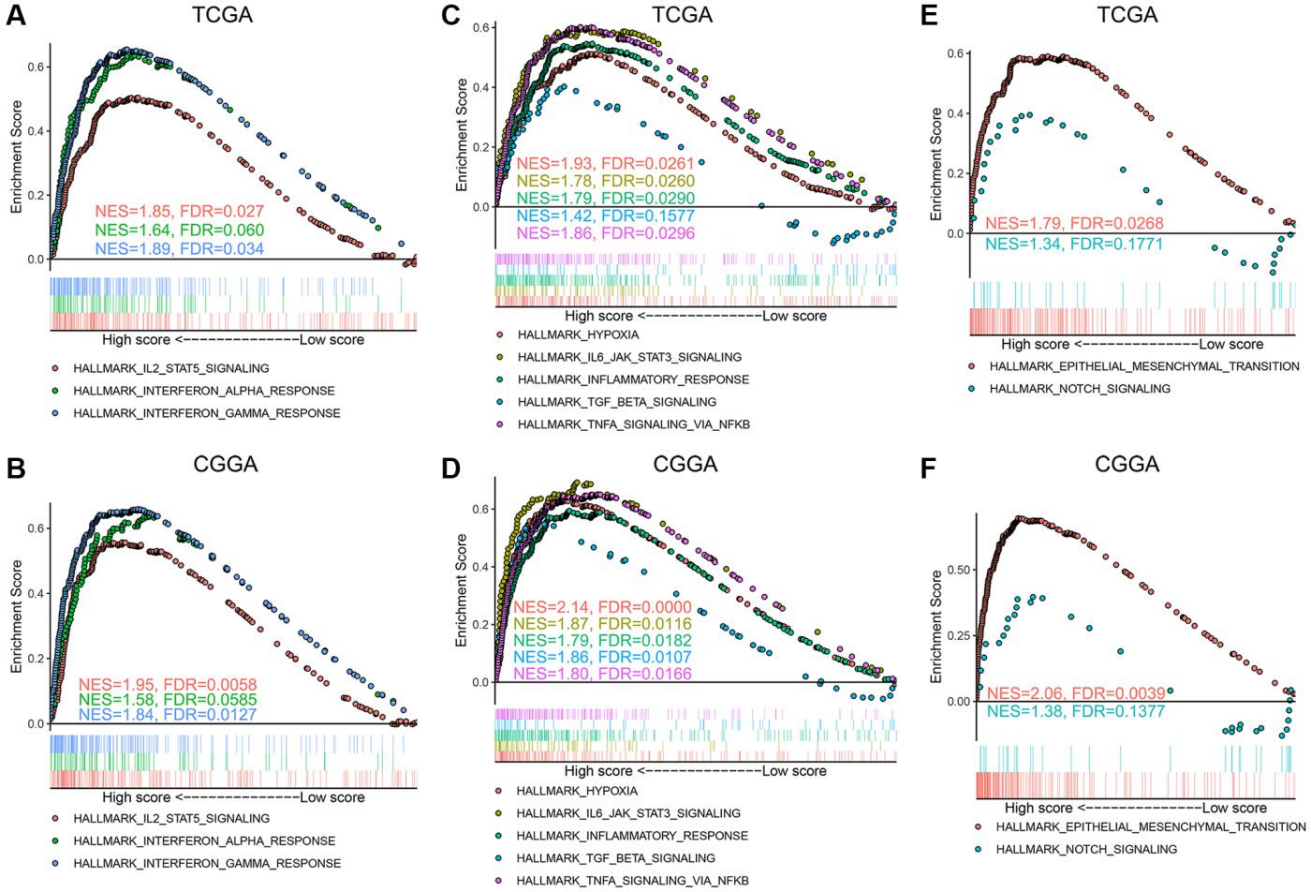
**Hallmark gene sets enriched in the high angiogenesis score group.** (**A**, **B**) Immune response related gene sets, including IL2-STAT5 signaling, interferon (IFN)-α and IFN-γ response, were enriched in the high-score gliomas in the TCGA (**A**) and CGGA (**B**) datasets. (**C**, **D**) Hypoxia, IL6-JAK-STAT3 signaling, inflammatory response, TGF-β signaling, and TNF-α signaling via NFkB gene sets were enriched in the high-score gliomas in the TCGA (**C**) and CGGA (**D**) datasets. (**E**, **F**) Epithelial mesenchymal transition (EMT) and NOTCH signaling gene sets were highly associated with high angiogenesis score in the TCGA (**E**) and CGGA (**F**) cohorts.

To further explore the significance of the angiogenesis pathway score in glioma, we conducted a GSEA analysis of the Hallmark gene sets in the high- and low-score groups. Immune response-related gene sets, such as IL2-STAT5 signaling, interferon (IFN)-α and IFN-γ responses, were significantly enriched in the high-score glioma group in TCGA, CGGA, and CGGA (array) datasets (Figure 5A, 5B, Supplementary Figure 5A), which confirmed that the angiogenesis pathway score was closely associated with the anti-glioma immune response. In addition, unfavorable gene sets including hypoxia, IL6-JAK-STAT3 signaling, inflammatory response, TGF-β signaling, and TNF-signaling via NFkB gene sets were also highly enriched in the high-score groups in the three datasets (Figure 5C–5D, Supplementary Figure 5B), which explained the poor prognosis of the high-score glioma group from the perspective of signaling pathways.

Furthermore, we also found that the enrichment levels of epithelial mesenchymal transition (EMT) and NOTCH signaling in the high-score group were significantly higher than those in the low-score group in the TCGA, CGGA, and CGGA (array) datasets (Figure 5E, 5F, Supplementary Figure 5C). Furthermore, in our sequencing results, the angiogenesis score had a strong positive correlation with the EMT score, suggesting that the tumors in the high-scoring group had a stronger predisposition to EMT (Supplementary Figure 5D). Considering the vital role of EMT and NOTCH signaling in glioma metastasis [37, 38], we believe that the angiogenesis pathway score indicated the malignant phenotype of glioma from the perspective of glioma metastasis. These findings indicate that high intra-glioma angiogenesis is correlated with hypoxia, unfavorable inflammation, and glioma metastasis.

### Glioma angiogenesis score was associated with chemotherapy

**Figure 6:**
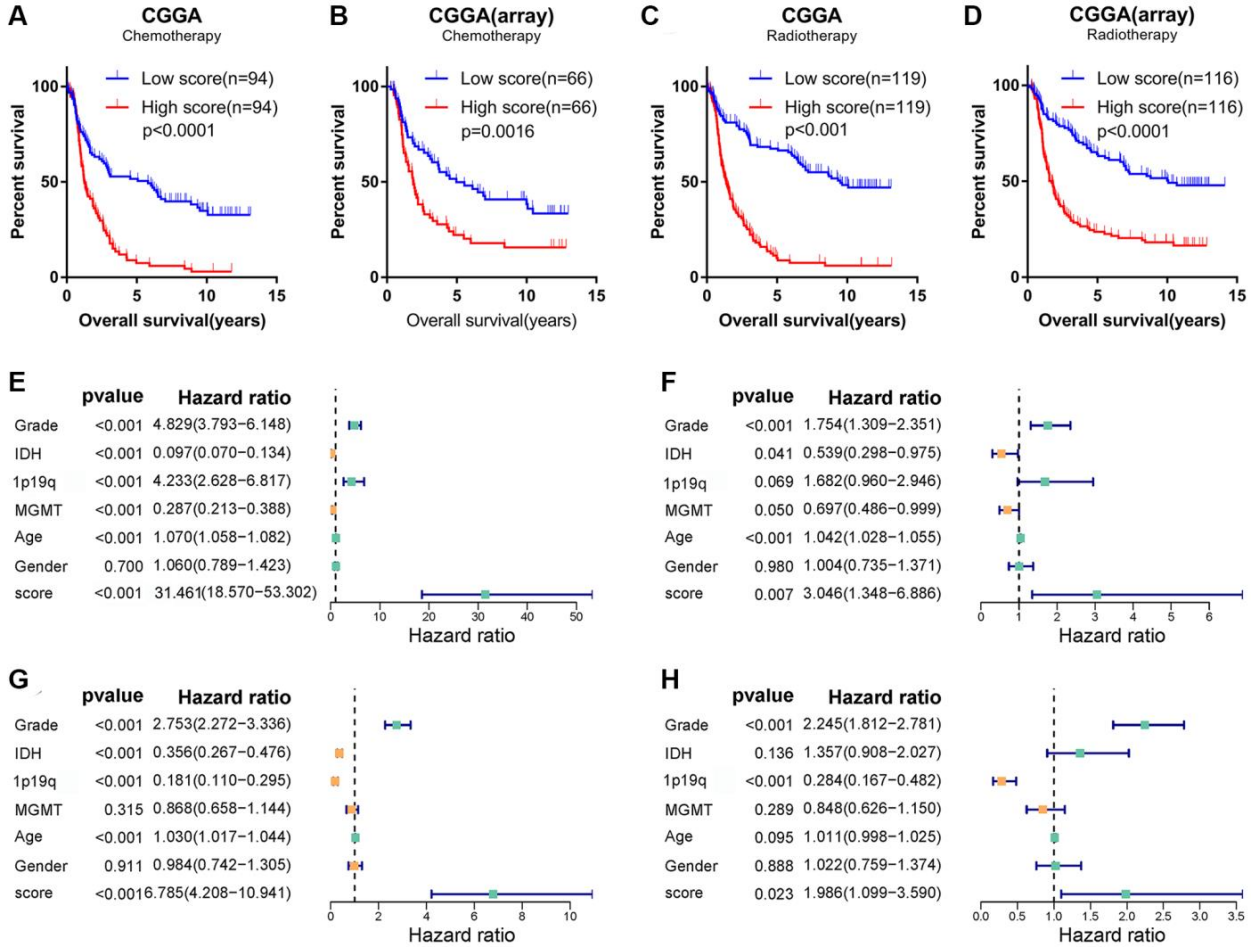
**Angiogenesis score, as an independent prognostic factor, reflected glioma sensitivity to therapy.** (**A**–**D**) Among glioma patients receiving chemotherapy (**A**, **B**) or radiotherapy (**C**, **D**), the prognosis of glioma patients in the high-score group was significantly worse than that in the low-score group in the CGGA and CGGA (array) datasets. (**E**–**H**) Univariate (**E**, **G**) and multivariate (**F**, **H**) Cox regression analysis in the TCGA (**E**, **F**) and CGGA (**G**, **H**) datasets revealed that angiogenesis score was an independent prognostic factor for glioma patients.

Despite compelling effects in other cancers, the outcomes of anti-angiogenic therapies, such as bevacizumab, and cilengitide, were disappointing in gliomas [26]. Gliomas are easily resistant to those agents. In addition, gliomas tend to be resistant to radiotherapy and chemotherapy. Considering the relationship between the angiogenesis pathway score and the malignant phenotype of glioma, we speculated that the score might be related to the treatment tolerance of glioma. Thus, we further explored the prognosis of glioma patients who received different treatments in CGGA and CGGA (array) cohorts. Interestingly, in glioma patients who received radiotherapy or chemotherapy, the OS of the high-score group was significantly shorter than that of the low-score group (Figure 6A–6D). We analyzed LGG and GBM separately and found that the score can distinguish the prognosis of patients receiving different treatments (Supplementary Figure 6A–6H). Univariate and multivariate COX regression analysis also found that the score is an independent prognostic factor for glioma patients receiving chemotherapy or radiotherapy (Supplementary Figure 7A–7H). To develop a therapy target, it is important to analyse the correlation between angiogenesis score and existing drugs. In our study, drug-sensitivity analysis revealed that angiogenesis score was positively correlated with Axitinib, Imatinib, Vorinostat and negatively correlated with Cetuximab, Cisplatin and Docetaxel in the cancer therapeutic response portal database (Supplementary Figure 8). These results show that the angiogenesis score can not only accurately predict the prognosis of glioma patients, but also show the potential of guiding chemotherapy dosing.

Since the angiogenesis pathway score has such a powerful potential in predicting the prognosis of glioma, we believed it may be an independent prognostic factor for glioma. To confirm this conjecture, univariate and multivariate Cox regression analyses were conducted in the TCGA, CGGA, and CGGA (array) databases to analyze the independence of angiogenesis pathway score in the prognosis of glioma. As shown in Figure 6E–6H and Supplementary Figure 7I, 7J, the angiogenesis pathway score was independent of other clinicopathological features and acted as a risk factor for the prognosis of glioma.

## DISCUSSION

In this study, we explored the angiogenesis pathway score, which was measured by GSVA score based on the “HALLMARK_ ANGIOGENESIS” gene set retrieved from MSigDb, in TCGA, CGGA, CGGA (array), and GSE16011 datasets. This was done to reveal the relationship between intra-tumoral angiogenesis in glioma and features such as cancer aggressiveness, immune microenvironment, metastasis, and treatment response. To the best of our knowledge, utilizing GSAV score to explore the clinical relevance of intra-tumoral angiogenesis in glioma has not been previously reported.

The angiogenesis pathway score correlated with significantly enriched expression for VEGF-related genes, vascular endothelial cell markers, vascular stability related- genes and hypoxia-related genes. This indicates the score can accurately assess the status of intra-tumoral angiogenesis in glioma. The prognosis of glioma patients with high angiogenesis pathway score was significantly worse than that of low-score patients. High angiogenesis pathway score gliomas demonstrated a predilection for aggressive clinicopathological characteristics, such as higher grade, IDH wildtype genotype, 1p19q non-codeletion, MGMT promoter unmethylated status, and mesenchymal subtype. In addition, high score correlated with lower tumor purity and greater immune cell infiltration, regardless of pro- or anti-cancerous immune cells, and also higher expression of most immune checkpoints. The angiogenesis pathway score decreased as glioma sensitivity to radiotherapy and chemotherapy increased. Furthermore, the angiogenesis pathway score acted as an independent prognostic factor to accurately predict the outcome of glioma patients.

The structure of vessels in tumor is dissimilar from that in normal tissue. Blood vessels in tumor branch irregularly, follow a crisscross pattern, are highly permeable, and are often larger with irregular luminal diameter [5]. It is precisely because of these characteristics, that the conclusions in our study can, at least, partially be explained. Regulation of angiogenesis involves multi-level signal transduction. A typical example of this is the hypermetabolic state of the tumor cells leading to local hypoxia, which in turn activates HIF-1. HIF1 then promotes angiogenesis through VEGF signaling [39, 40]. This coincides with the difference in expression of related genes in the high- and low-score groups and confirms that the angiogenesis pathway score can predict intra-tumoral angiogenesis status. In addition, hemodynamic changes caused by changes in vascular structure affect blood flow and flux of leukocytes within the tumor [41]. This is also consistent with the relationship between the angiogenesis score and immune cell infiltration within the glioma microenvironment. Moreover, the incompleteness of tumor vasculature, with defective basement membrane being one example of this, provides a path for tumor cell metastasis [42]. Perhaps this is one of the reasons why the EMT and NOTCH signaling pathways are enriched in the high-score glioma group.

Tumor angiogenesis is not only affected by tumor cells. Other cells in the tumor microenvironment also have a regulatory effect. For instance, tumor-associated macrophages participate in all stages of angiogenesis in gliomas by releasing paracrine factors such as WNT7b and M-CSF [43]. This underscores the idea that regulation of tumor angiogenesis and the influence of tumor blood vessels on tumors is highly complicated. Given this, the obvious shortcoming of our study is that although we have found the angiogenesis pathway score can be used as an independent prognostic factor to reflect the malignant phenotype and immune characteristics of glioma, the specific underlying mechanisms are unclear and further investigation is warranted to explore them.

In conclusion, our study revealed that the angiogenesis pathway score, as an independent prognostic factor, can distinguish the malignant phenotype of glioma and accurately predict the immune characteristics and prognosis of glioma.

## MATERIALS AND METHODS

### Human specimens

The present study has been reviewed and approved by the Institutional Review Board (IRB) of the Sheng Jing hospital of China Medical University, and all patients have provided their written informed consent. A total of 8 samples were collected and sent for bulk RNA sequencing. RNA sequencing was performed by the Illumina Next Generation Sequencing Service at the Beijing Genomics Institution in Shen Zhen. The diagnosis of glioma in each patient was rendered by integrated evaluation of preoperative imaging and postoperative pathology.

### Study cohort

The Cancer Genome Atlas (TCGA) glioma gene expression profile and corresponding clinical information were retrieved from TCGA database (http://cancergenome.nih.gov/). The Chinese Glioma Genome Atlas (CGGA) mRNA expression data (mRNAseq_325 and mRNA-array) and relevant clinicopathological information were obtained from the CGGA database (http://www.cgga.org.cn) (CGGA will be used to refer to the CGGA mRNAseq_325 dataset unless otherwise specified). The GSE16011 dataset was retrieved from the Gene Expression Omnibus (GEO) database (http://www.ncbi.nlm.nih.gov/geo). The background correction and normalization of the GSE16011 raw data was conducted using the robust multi-array analysis (RMA) method of the affy R package. Sample characteristics, including detailed data for each research object, are shown in Supplementary Table 1.

### Gene set expression analyses (GSEA)

The HALLMARK_ANGIOGENESIS gene set, which was acquired from hallmark gene sets of molecular signatures database (MSigDb) (v7.2), was employed to calculate the angiogenesis pathway score using the GSVA package in R. Gene Set Enrichment Analysis (GSEA) was conducted using GSEA software (v4.1.0) and the GSEA results with false discovery rate (FDR) <0.25 were considered as statistically significant [44, 45].

### Estimation of stromal and immune cells content in tumor tissues

The ESTIMATE R package is designed to infer tumor purity and the presence of infiltrating stromal/immune cells within tumor tissues using gene expression data [46]. We used the ESTIMATE R package to measure the stromal score, immune score, and tumor purity of glioma.

### Immune cell enrichment analysis

The single-sample GSEA (ssGSEA) method of *GSVA* package in R can identify the enrichment of immune cells within tumor microenvironment using the gene expression signatures [47]. We used this method to measure the enrichment level of immune cells in glioma samples.

### Prognosis analyses

Glioma samples were divided into high- and low-score groups according to the median angiogenesis pathway score. The overall survival (OS) difference between the high- and low-score groups was determined using the Kaplan-Meier (KM) method of the survival R package. Univariate and multivariate Cox regression analyses were conducted using the survival package to assess the independence of the angiogenesis pathway score in predicting the OS of glioma patients.

### Drug susceptibility analysis

We calculated the semi-inhibitory concentration (IC50) values of chemotherapeutic drugs using the “pRRophetic” package.

### Statistical analysis

The R software (version 4.0.2) and GraphPad Prism (version 7.0.0) were used for most statistical analyses and graphing in this study. The R package “limma” was used to identify the difference of immune cells enrichment scores and the expression of immune check points between high- and low-score groups. Wilcoxon signed-rank test was used to compare gene expression between two groups while Mann-Whitney *U* test was applied for multiple groups. The correlation of two variables was accessed by Pearson’s correlation test. *P* value < 0.05 was considered statistically significant. ^*^p < 0.05, ^**^p < 0.01, ^***^p < 0.001, ^****^p < 0.0001.

### Data availability statement

The datasets used in this study can be downloaded from: http://www.cgga.org.cn/ (CGGA), https://portal.gdc.cancer.gov/ (TCGA), and https://www.ncbi.nlm.nih.gov/geo/query/acc.cgi?acc=GSE16011 (GSE16011).

## Supplementary Materials

Supplementary Figures

Supplementary Table 1

## References

[1] Louis DN, Perry A, Reifenberger G, von Deimling A, Figarella-Branger D, Cavenee WK, Ohgaki H, Wiestler OD, Kleihues P, Ellison DW. The 2016 World Health Organization Classification of Tumors of the Central Nervous System: a summary. Acta Neuropathol. 2016; 131:803–20. 10.1007/s00401-016-1545-1 27157931

[2] Lin C, Lin L, Mao S, Yang L, Yi L, Lin X, Wang J, Lin ZX, Lin JM. Reconstituting Glioma Perivascular Niches on a Chip for Insights into Chemoresistance of Glioma. Anal Chem. 2018; 90:10326–33. 10.1021/acs.analchem.8b02133 30094990

[3] Stupp R, Hegi ME, Mason WP, van den Bent MJ, Taphoorn MJ, Janzer RC, Ludwin SK, Allgeier A, Fisher B, Belanger K, Hau P, Brandes AA, Gijtenbeek J, et al, and European Organisation for Research and Treatment of Cancer Brain Tumour and Radiation Oncology Groups, and National Cancer Institute of Canada Clinical Trials Group. Effects of radiotherapy with concomitant and adjuvant temozolomide versus radiotherapy alone on survival in glioblastoma in a randomised phase III study: 5-year analysis of the EORTC-NCIC trial. Lancet Oncol. 2009; 10:459–66. 10.1016/S1470-2045(09)70025-7 19269895

[4] Folkman J. Tumor angiogenesis: therapeutic implications. N Engl J Med. 1971; 285:1182–6. 10.1056/NEJM197111182852108 4938153

[5] Viallard C, Larrivée B. Tumor angiogenesis and vascular normalization: alternative therapeutic targets. Angiogenesis. 2017; 20:409–26. 10.1007/s10456-017-9562-9 28660302

[6] Gupta MK, Qin RY. Mechanism and its regulation of tumor-induced angiogenesis. World J Gastroenterol. 2003; 9:1144–55. 10.3748/wjg.v9.i6.1144 12800214 PMC4611774

[7] De Palma M, Biziato D, Petrova TV. Microenvironmental regulation of tumour angiogenesis. Nat Rev Cancer. 2017; 17:457–74. 10.1038/nrc.2017.51 28706266

[8] Priceman SJ, Sung JL, Shaposhnik Z, Burton JB, Torres-Collado AX, Moughon DL, Johnson M, Lusis AJ, Cohen DA, Iruela-Arispe ML, Wu L. Targeting distinct tumor-infiltrating myeloid cells by inhibiting CSF-1 receptor: combating tumor evasion of antiangiogenic therapy. Blood. 2010; 115:1461–71. 10.1182/blood-2009-08-237412 20008303 PMC2826767

[9] Stockmann C, Doedens A, Weidemann A, Zhang N, Takeda N, Greenberg JI, Cheresh DA, Johnson RS. Deletion of vascular endothelial growth factor in myeloid cells accelerates tumorigenesis. Nature. 2008; 456:814–8. 10.1038/nature07445 18997773 PMC3103772

[10] Lewis JS, Landers RJ, Underwood JC, Harris AL, Lewis CE. Expression of vascular endothelial growth factor by macrophages is up-regulated in poorly vascularized areas of breast carcinomas. J Pathol. 2000; 192:150–8. 10.1002/1096-9896(2000)9999:9999<::AID-PATH687>3.0.CO;2-G 11004690

[11] Maishi N, Hida K. Tumor endothelial cells accelerate tumor metastasis. Cancer Sci. 2017; 108:1921–6. 10.1111/cas.13336 28763139 PMC5623747

[12] Fox SB, Leek RD, Weekes MP, Whitehouse RM, Gatter KC, Harris AL. Quantitation and prognostic value of breast cancer angiogenesis: comparison of microvessel density, Chalkley count, and computer image analysis. J Pathol. 1995; 177:275–83. 10.1002/path.1711770310 8551390

[13] Weidner N, Carroll PR, Flax J, Blumenfeld W, Folkman J. Tumor angiogenesis correlates with metastasis in invasive prostate carcinoma. Am J Pathol. 1993; 143:401–9. 7688183 PMC1887042

[14] Giatromanolaki A, Koukourakis M, O'Byrne K, Fox S, Whitehouse R, Talbot DC, Harris AL, Gatter KC. Prognostic value of angiogenesis in operable non-small cell lung cancer. J Pathol. 1996; 179:80–8. 10.1002/(SICI)1096-9896(199605)179:1<80::AID-PATH547>3.0.CO;2-X 8691350

[15] Brustmann H, Riss P, Naudé S. The relevance of angiogenesis in benign and malignant epithelial tumors of the ovary: a quantitative histologic study. Gynecol Oncol. 1997; 67:20–6. 10.1006/gyno.1997.4815 9345351

[16] Brantley-Sieders DM, Dunaway CM, Rao M, Short S, Hwang Y, Gao Y, Li D, Jiang A, Shyr Y, Wu JY, Chen J. Angiocrine factors modulate tumor proliferation and motility through EphA2 repression of Slit2 tumor suppressor function in endothelium. Cancer Res. 2011; 71:976–87. 10.1158/0008-5472.CAN-10-3396 21148069 PMC3032824

[17] Melder RJ, Koenig GC, Witwer BP, Safabakhsh N, Munn LL, Jain RK. During angiogenesis, vascular endothelial growth factor and basic fibroblast growth factor regulate natural killer cell adhesion to tumor endothelium. Nat Med. 1996; 2:992–7. 10.1038/nm0996-992 8782456

[18] Dirkx AE, Oude Egbrink MG, Kuijpers MJ, van der Niet ST, Heijnen VV, Bouma-ter Steege JC, Wagstaff J, Griffioen AW. Tumor angiogenesis modulates leukocyte-vessel wall interactions in vivo by reducing endothelial adhesion molecule expression. Cancer Res. 2003; 63:2322–9. 12727857

[19] Tate MC, Aghi MK. Biology of angiogenesis and invasion in glioma. Neurotherapeutics. 2009; 6:447–57. 10.1016/j.nurt.2009.04.001 19560735 PMC5084181

[20] Broekman ML, Maas SLN, Abels ER, Mempel TR, Krichevsky AM, Breakefield XO. Multidimensional communication in the microenvirons of glioblastoma. Nat Rev Neurol. 2018; 14:482–95. 10.1038/s41582-018-0025-8 29985475 PMC6425928

[21] Jain RK. Antiangiogenesis strategies revisited: from starving tumors to alleviating hypoxia. Cancer Cell. 2014; 26:605–22. 10.1016/j.ccell.2014.10.006 25517747 PMC4269830

[22] Plate KH, Scholz A, Dumont DJ. Tumor angiogenesis and anti-angiogenic therapy in malignant gliomas revisited. Acta Neuropathol. 2012; 124:763–75. 10.1007/s00401-012-1066-5 23143192 PMC3508273

[23] Carmeliet P, Jain RK. Molecular mechanisms and clinical applications of angiogenesis. Nature. 2011; 473:298–307. 10.1038/nature10144 21593862 PMC4049445

[24] Bergers G, Hanahan D. Modes of resistance to anti-angiogenic therapy. Nat Rev Cancer. 2008; 8:592–603. 10.1038/nrc2442 18650835 PMC2874834

[25] Sennino B, McDonald DM. Controlling escape from angiogenesis inhibitors. Nat Rev Cancer. 2012; 12:699–709. 10.1038/nrc3366 23001349 PMC3969886

[26] Wick W, Platten M, Wick A, Hertenstein A, Radbruch A, Bendszus M, Winkler F. Current status and future directions of anti-angiogenic therapy for gliomas. Neuro Oncol. 2016; 18:315–28. 10.1093/neuonc/nov180 26459812 PMC4767238

[27] Carmeliet P, Jain RK. Angiogenesis in cancer and other diseases. Nature. 2000; 407:249–57. 10.1038/35025220 11001068

[28] von Tell D, Armulik A, Betsholtz C. Pericytes and vascular stability. Exp Cell Res. 2006; 312:623–9. 10.1016/j.yexcr.2005.10.019 16303125

[29] Choudhry H, Harris AL. Advances in Hypoxia-Inducible Factor Biology. Cell Metab. 2018; 27:281–98. 10.1016/j.cmet.2017.10.005 29129785

[30] Eckel-Passow JE, Lachance DH, Molinaro AM, Walsh KM, Decker PA, Sicotte H, Pekmezci M, Rice T, Kosel ML, Smirnov IV, Sarkar G, Caron AA, Kollmeyer TM, et al. Glioma Groups Based on 1p/19q, IDH, and TERT Promoter Mutations in Tumors. N Engl J Med. 2015; 372:2499–508. 10.1056/NEJMoa1407279 26061753 PMC4489704

[31] Verhaak RG, Hoadley KA, Purdom E, Wang V, Qi Y, Wilkerson MD, Miller CR, Ding L, Golub T, Mesirov JP, Alexe G, Lawrence M, O'Kelly M, et al, and Cancer Genome Atlas Research Network. Integrated genomic analysis identifies clinically relevant subtypes of glioblastoma characterized by abnormalities in PDGFRA, IDH1, EGFR, and NF1. Cancer Cell. 2010; 17:98–110. 10.1016/j.ccr.2009.12.020 20129251 PMC2818769

[32] Zhang C, Cheng W, Ren X, Wang Z, Liu X, Li G, Han S, Jiang T, Wu A. Tumor Purity as an Underlying Key Factor in Glioma. Clin Cancer Res. 2017; 23:6279–91. 10.1158/1078-0432.CCR-16-2598 28754819

[33] Pardoll DM. The blockade of immune checkpoints in cancer immunotherapy. Nat Rev Cancer. 2012; 12:252–64. 10.1038/nrc3239 22437870 PMC4856023

[34] Larkin J, Chiarion-Sileni V, Gonzalez R, Grob JJ, Cowey CL, Lao CD, Schadendorf D, Dummer R, Smylie M, Rutkowski P, Ferrucci PF, Hill A, Wagstaff J, et al. Combined Nivolumab and Ipilimumab or Monotherapy in Untreated Melanoma. N Engl J Med. 2015; 373:23–34. 10.1056/NEJMoa1504030 26027431 PMC5698905

[35] Borghaei H, Paz-Ares L, Horn L, Spigel DR, Steins M, Ready NE, Chow LQ, Vokes EE, Felip E, Holgado E, Barlesi F, Kohlhäufl M, Arrieta O, et al. Nivolumab versus Docetaxel in Advanced Nonsquamous Non-Small-Cell Lung Cancer. N Engl J Med. 2015; 373:1627–39. 10.1056/NEJMoa1507643 26412456 PMC5705936

[36] Motzer RJ, Escudier B, McDermott DF, George S, Hammers HJ, Srinivas S, Tykodi SS, Sosman JA, Procopio G, Plimack ER, Castellano D, Choueiri TK, Gurney H, et al, and CheckMate 025 Investigators. Nivolumab versus Everolimus in Advanced Renal-Cell Carcinoma. N Engl J Med. 2015; 373:1803–13. 10.1056/NEJMoa1510665 26406148 PMC5719487

[37] Wang J, Xu SL, Duan JJ, Yi L, Guo YF, Shi Y, Li L, Yang ZY, Liao XM, Cai J, Zhang YQ, Xiao HL, Yin L, et al. Invasion of white matter tracts by glioma stem cells is regulated by a NOTCH1-SOX2 positive-feedback loop. Nat Neurosci. 2019; 22:91–105. 10.1038/s41593-018-0285-z 30559479

[38] Lah TT, Novak M, Breznik B. Brain malignancies: Glioblastoma and brain metastases. Semin Cancer Biol. 2020; 60:262–73. 10.1016/j.semcancer.2019.10.010 31654711

[39] Semenza GL. Hypoxia-inducible factor 1: oxygen homeostasis and disease pathophysiology. Trends Mol Med. 2001; 7:345–50. 10.1016/s1471-4914(01)02090-1 11516994

[40] Neufeld G, Cohen T, Gengrinovitch S, Poltorak Z. Vascular endothelial growth factor (VEGF) and its receptors. FASEB J. 1999; 13:9–22. 9872925

[41] Folkins C, Shaked Y, Man S, Tang T, Lee CR, Zhu Z, Hoffman RM, Kerbel RS. Glioma tumor stem-like cells promote tumor angiogenesis and vasculogenesis via vascular endothelial growth factor and stromal-derived factor 1. Cancer Res. 2009; 69:7243–51. 10.1158/0008-5472.CAN-09-0167 19738068 PMC3409689

[42] Papetti M, Herman IM. Mechanisms of normal and tumor-derived angiogenesis. Am J Physiol Cell Physiol. 2002; 282:C947–70. 10.1152/ajpcell.00389.2001 11940508

[43] Zhu C, Kros JM, Cheng C, Mustafa D. The contribution of tumor-associated macrophages in glioma neo-angiogenesis and implications for anti-angiogenic strategies. Neuro Oncol. 2017; 19:1435–46. 10.1093/neuonc/nox081 28575312 PMC5737221

[44] Subramanian A, Tamayo P, Mootha VK, Mukherjee S, Ebert BL, Gillette MA, Paulovich A, Pomeroy SL, Golub TR, Lander ES, Mesirov JP. Gene set enrichment analysis: a knowledge-based approach for interpreting genome-wide expression profiles. Proc Natl Acad Sci U S A. 2005; 102:15545–50. 10.1073/pnas.0506580102 16199517 PMC1239896

[45] Oshi M, Katsuta E, Yan L, Ebos JML, Rashid OM, Matsuyama R, Endo I, Takabe K. A Novel 4-Gene Score to Predict Survival, Distant Metastasis and Response to Neoadjuvant Therapy in Breast Cancer. Cancers (Basel). 2020; 12:1148. 10.3390/cancers12051148 32370309 PMC7281399

[46] Yoshihara K, Shahmoradgoli M, Martínez E, Vegesna R, Kim H, Torres-Garcia W, Treviño V, Shen H, Laird PW, Levine DA, Carter SL, Getz G, Stemke-Hale K, et al. Inferring tumour purity and stromal and immune cell admixture from expression data. Nat Commun. 2013; 4:2612. 10.1038/ncomms3612 24113773 PMC3826632

[47] Charoentong P, Finotello F, Angelova M, Mayer C, Efremova M, Rieder D, Hackl H, Trajanoski Z. Pan-cancer Immunogenomic Analyses Reveal Genotype-Immunophenotype Relationships and Predictors of Response to Checkpoint Blockade. Cell Rep. 2017; 18:248–62. 10.1016/j.celrep.2016.12.019 28052254

