# Supplementary material for: Intra-tumoral angiogenesis correlates with immune features and prognosis in glioma
Source: Aging (Albany NY). 2022 May 17;14(10):4402–24. doi: 10.18632/aging.204079 (PMC9186765; doi:10.18632/aging.204079)
Supplement: Supplementary Figures [file aging-14-204079-s001.pdf]

SUPPLEMENTARY FIGURES

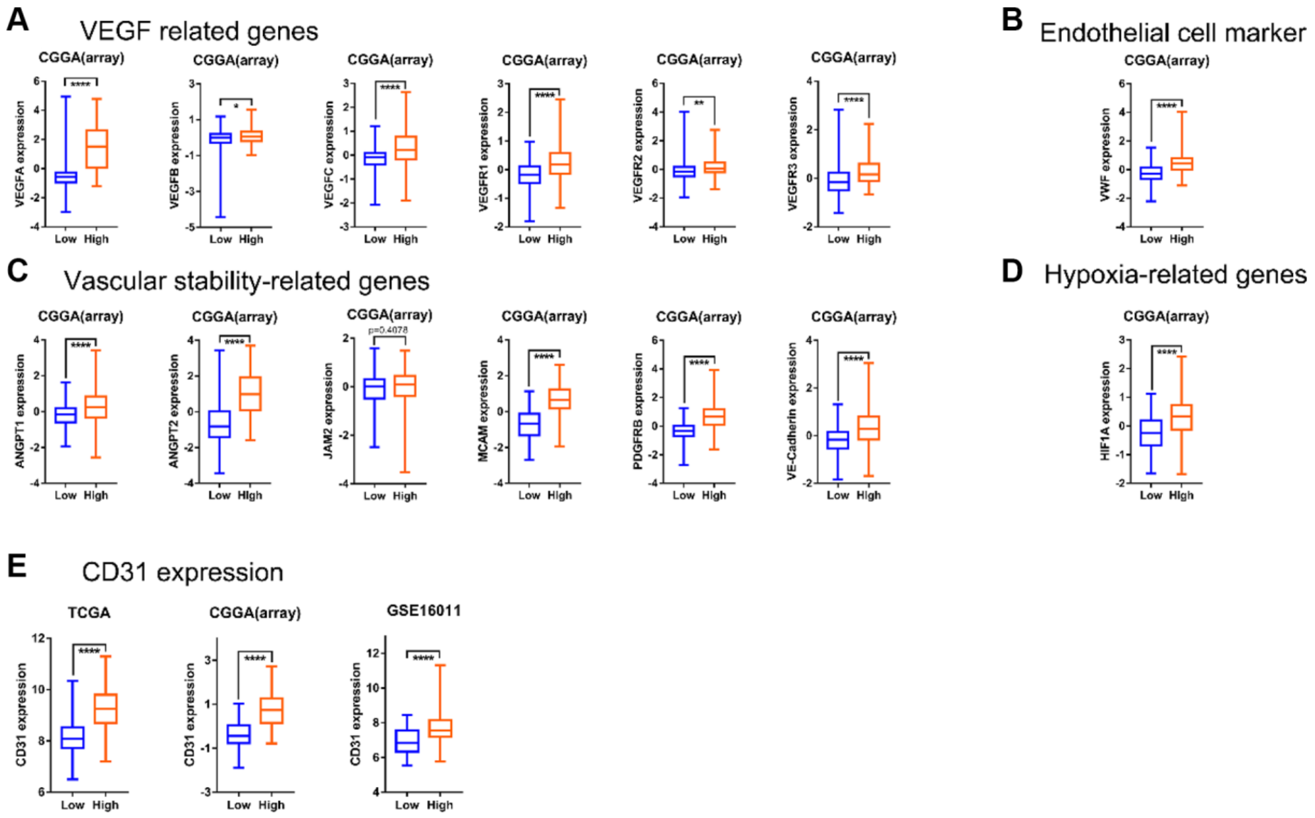

Supplementary Figure 1. The relationship between angiogenesis pathway score and angiogenesis regulatory factors in the CGGA (array) dataset. (**A**) Expression of VEGF-related genes (VEGFA, VEGFB, VEGFC, VEGFR1, VEGFR2, VEGFR3) in high- and low-score groups. (**B**) VWF (endothelial cell marker) expression in different groups. (**C**) Expression levels of ANGPT1, ANGPT2, JAM2, MCAM, PDGFRB, and VE-Cadherin in different groups. (**D**) Gene expression levels of hypoxia-related genes (HIF1A). (**E**) Expression of CD31 was significantly up-regulated in the high-score group in the TCGA, CGGA (array), and GSE16011 datasets.

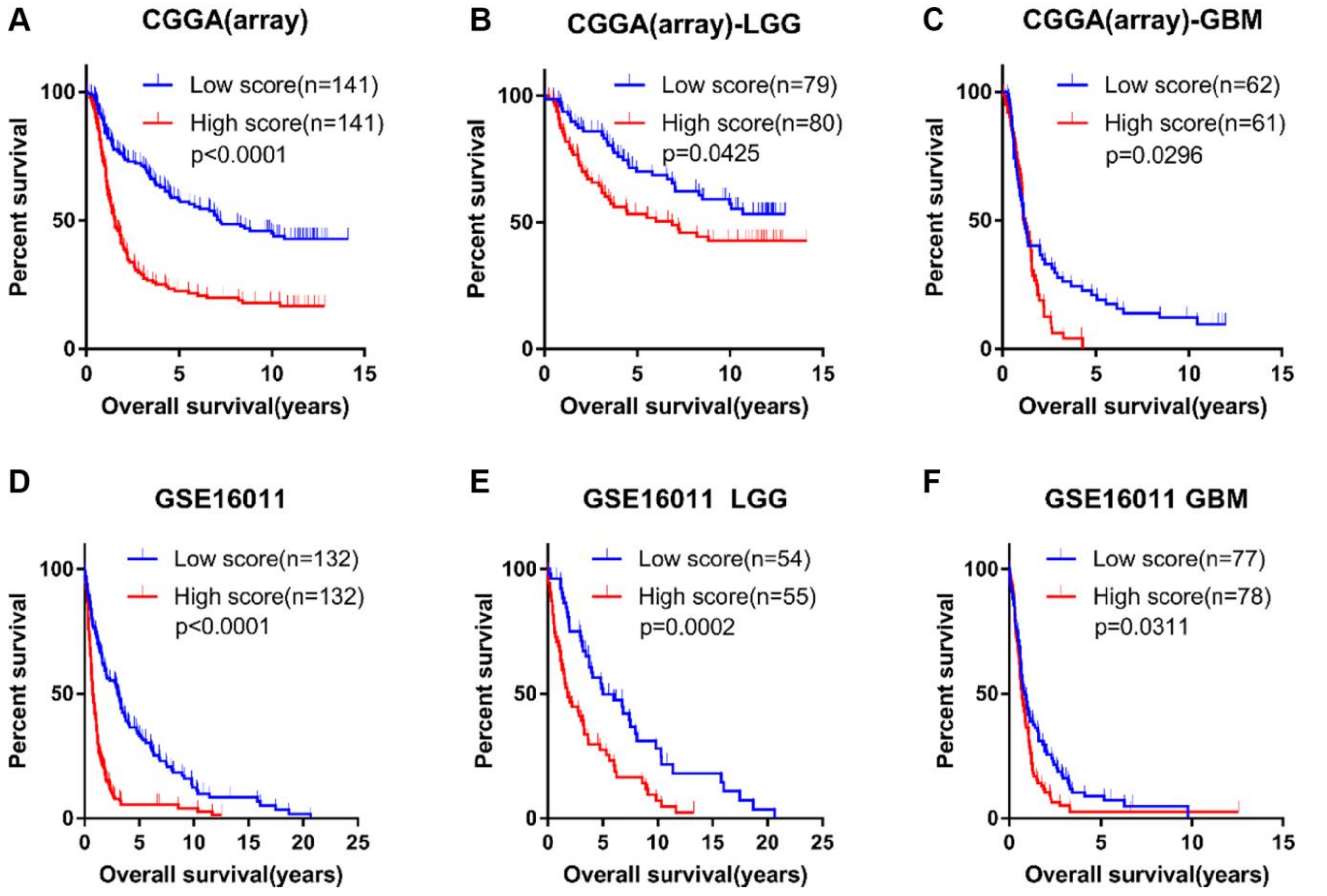

Supplementary Figure 2. Angiogenesis score accurately predicted the prognosis of glioma patients in the CGGA (array) and GSE16011 datasets. (**A**–**C**) The OS of high-score glioma (**A**), low grade glioma (LGG) (**B**) and glioblastoma (GBM) (**C**) patients was significantly shorter than that of the low-score group in the CGGA (array) cohorts. (**D**–**F**) The OS of high-score glioma (**D**), low grade glioma (LGG) (**E**) and glioblastoma (GBM) (**F**) patients was significantly shorter than that of the low-score group in GSE16011 cohorts.

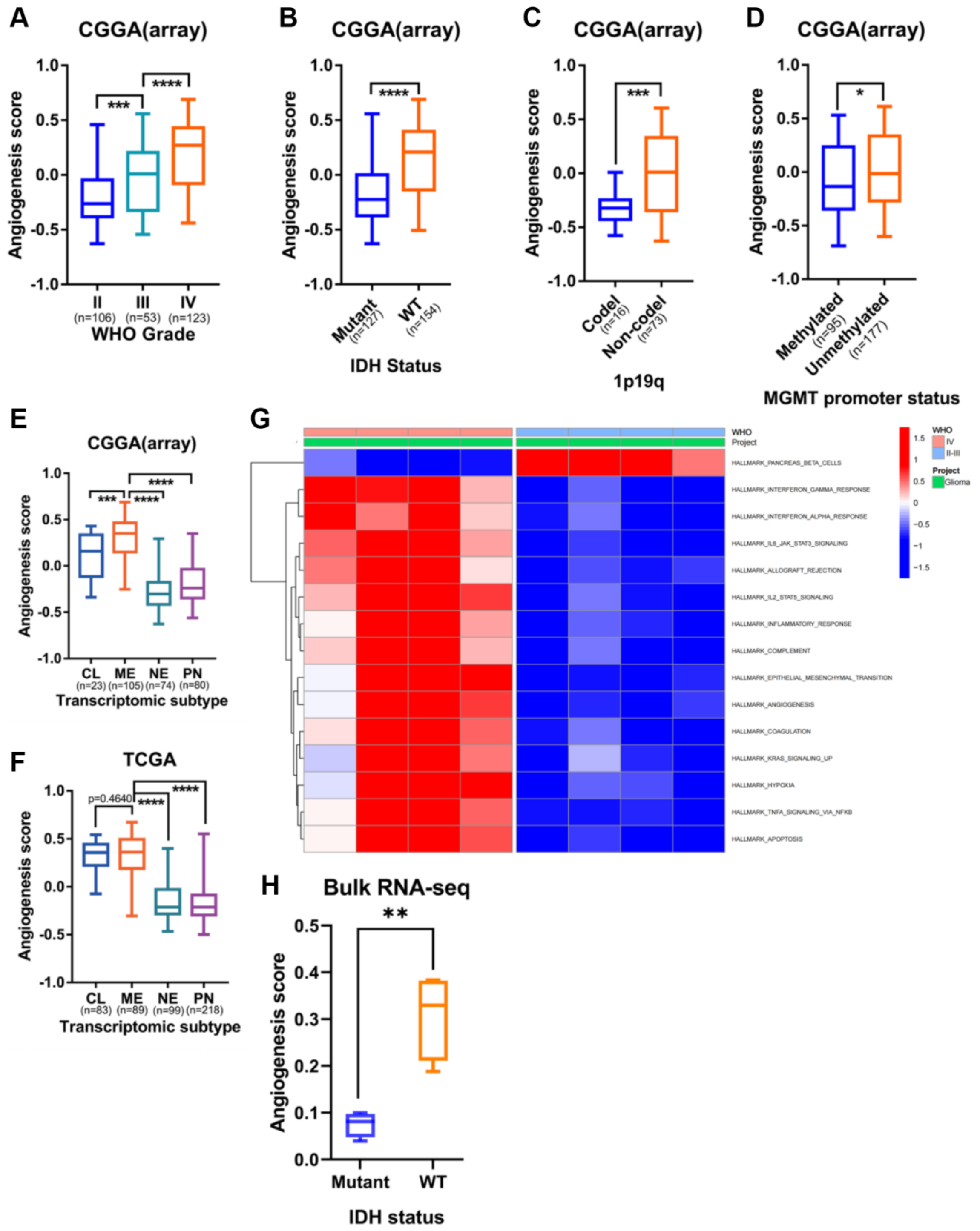

Supplementary Figure 3. Angiogenesis score distinguished the malignant subtypes of glioma in the CGGA (array) cohort. (**A**) The angiogenesis score was significantly correlated with WHO grade of glioma in the CGGA (array) (**A**) cohort. (**B**) Higher angiogenesis score in IDH-wildtype gliomas than in IDH-mutant gliomas. (**C**) Higher angiogenesis score in 1p19q non-codeleted gliomas than in 1p19q codeleted gliomas. (**D**) MGMT promoter unmethylated gliomas had a significantly higher angiogenesis score than MGMT promoter methylated gliomas. (**E**, **F**) The mesenchymal (ME) subtype had a significantly higher angiogenesis score than other transcriptome subtypes in the CGGA (array) (**E**) and TCGA (**F**) datasets. (**G**) GSVA analysis based on 8 patients' sequencing result. (**H**) Relationship between angiogenesis pathway score and IDH mutation.

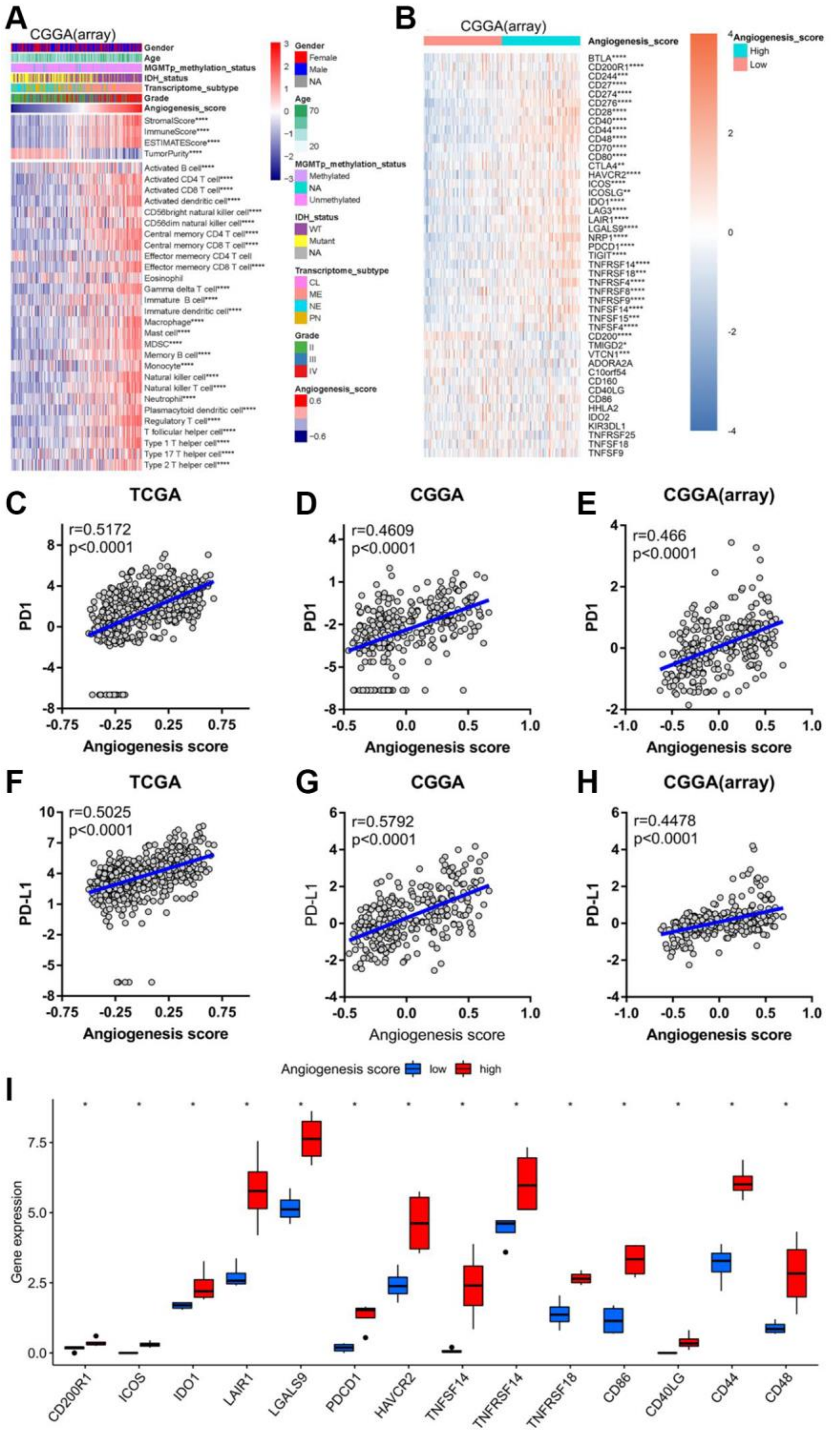

Supplementary Figure 4. The angiogenesis score reflected the immune characteristics of glioma in the CGGA (array) cohort. (**A**) With the increase of the angiogenesis pathway score, the purity of glioma was significantly reduced, the immune score was significantly increased, and the enrichment degree of most immune cells was also significantly increased. (**B**) The expression level of most immune checkpoints increased significantly as the angiogenesis score increased. (**C**–**E**) The correlation between PD-1 and angiogenesis score in TCGA, CGGA, and CGGA (array) datasets. (**F**–**H**) The correlation between PD-L1 and angiogenesis score in TCGA, CGGA, and CGGA (array) datasets. (**I**) Correlation between angiogenesis score and immune checkpoint gene expression in bulk RNA-seq.

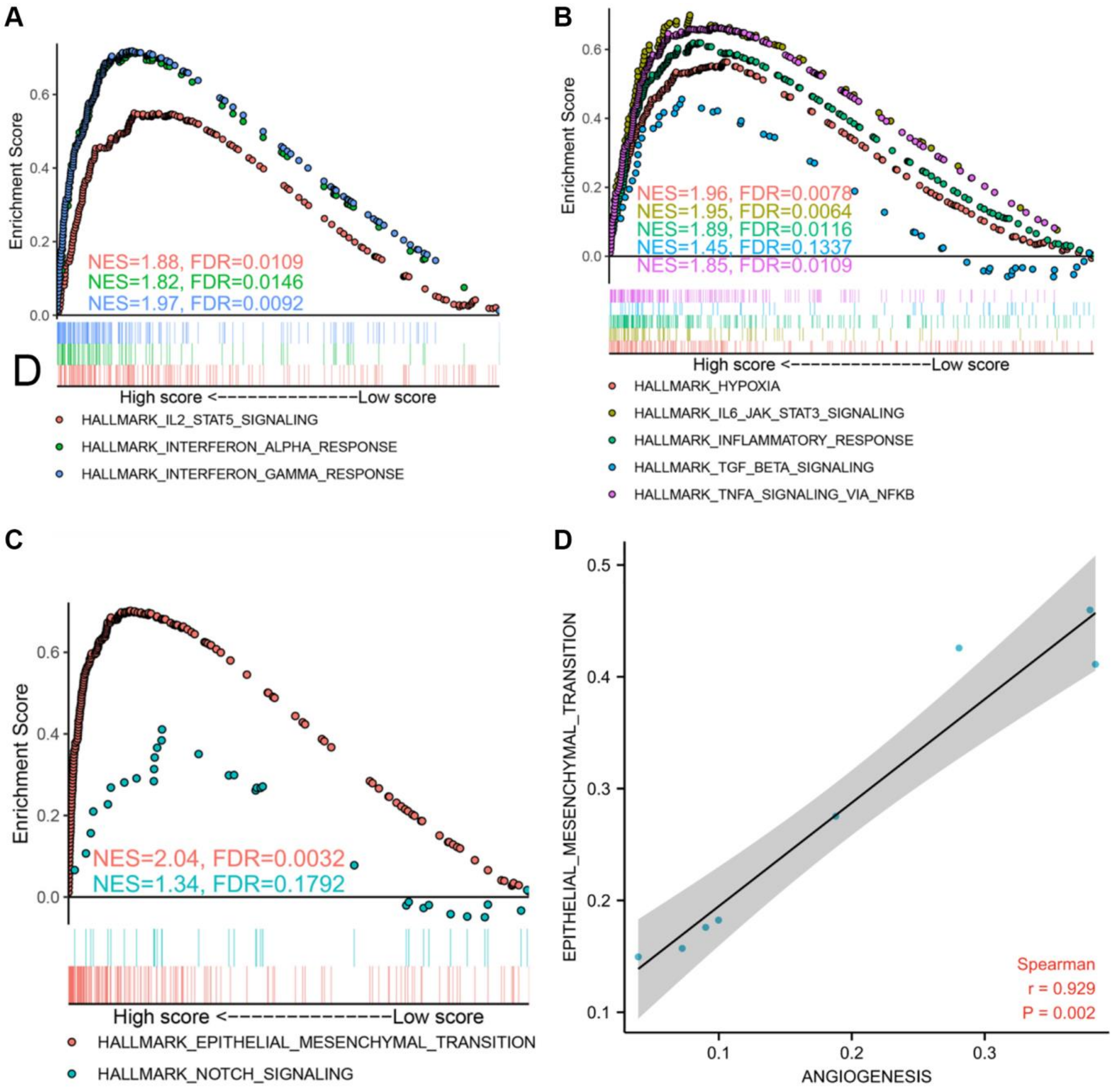

Supplementary Figure 5. Hallmark gene sets enriched in the high angiogenesis score group in the CGGA (array) cohort. (**A**) Immune response related gene sets, including IL2-STAT5 signaling, interferon (IFN)-α and IFN-γ response, were enriched in the high-score gliomas. (**B**) Hypoxia, IL6-JAK-STAT3 signaling, inflammatory response, TGF-β signaling, and TNF-α signaling via NFkB gene sets were enriched in the high-score gliomas. (**C**) Epithelial mesenchymal transition (EMT) and NOTCH signaling gene sets were highly associated with high angiogenesis score. (**D**) Correlation between angiogenesis score and EMT score.

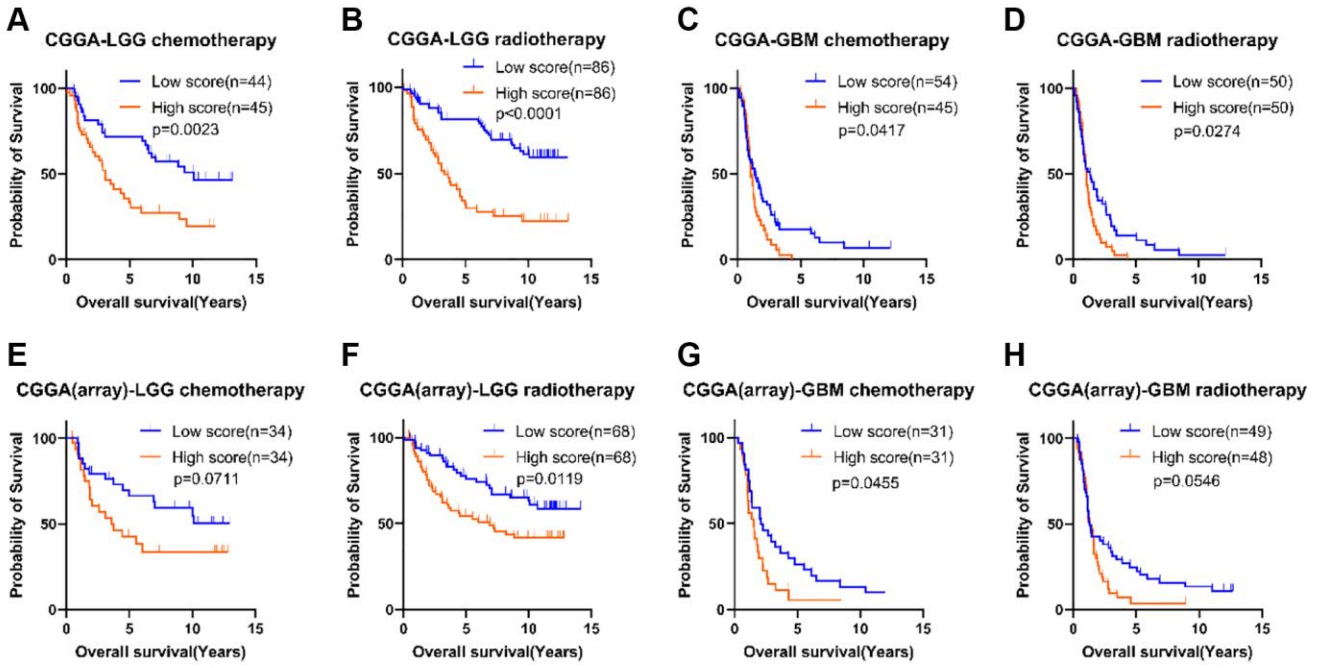

Supplementary Figure 6. Angiogenesis pathway score distinguished the prognosis of LGG and GBM patients receiving different treatment modalities. (**A**–**D**) In the CGGA cohort, the prognosis of LGG (**A**, **B**) and GBM (**C**, **D**) patients who received chemotherapy (**A**, **C**) and radiotherapy (**B**, **D**), respectively, in the high-scoring group was significantly worse than that in the low-scoring group. (**E**–**H**) In the CGGA_array cohorts, the prognosis of LGG (**E**, **F**) and GBM (**G**, **H**) patients who received chemotherapy (**E**, **G**) and radiotherapy (**F**, **H**), respectively, in the high-scoring group was significantly worse than that in the low-scoring group.

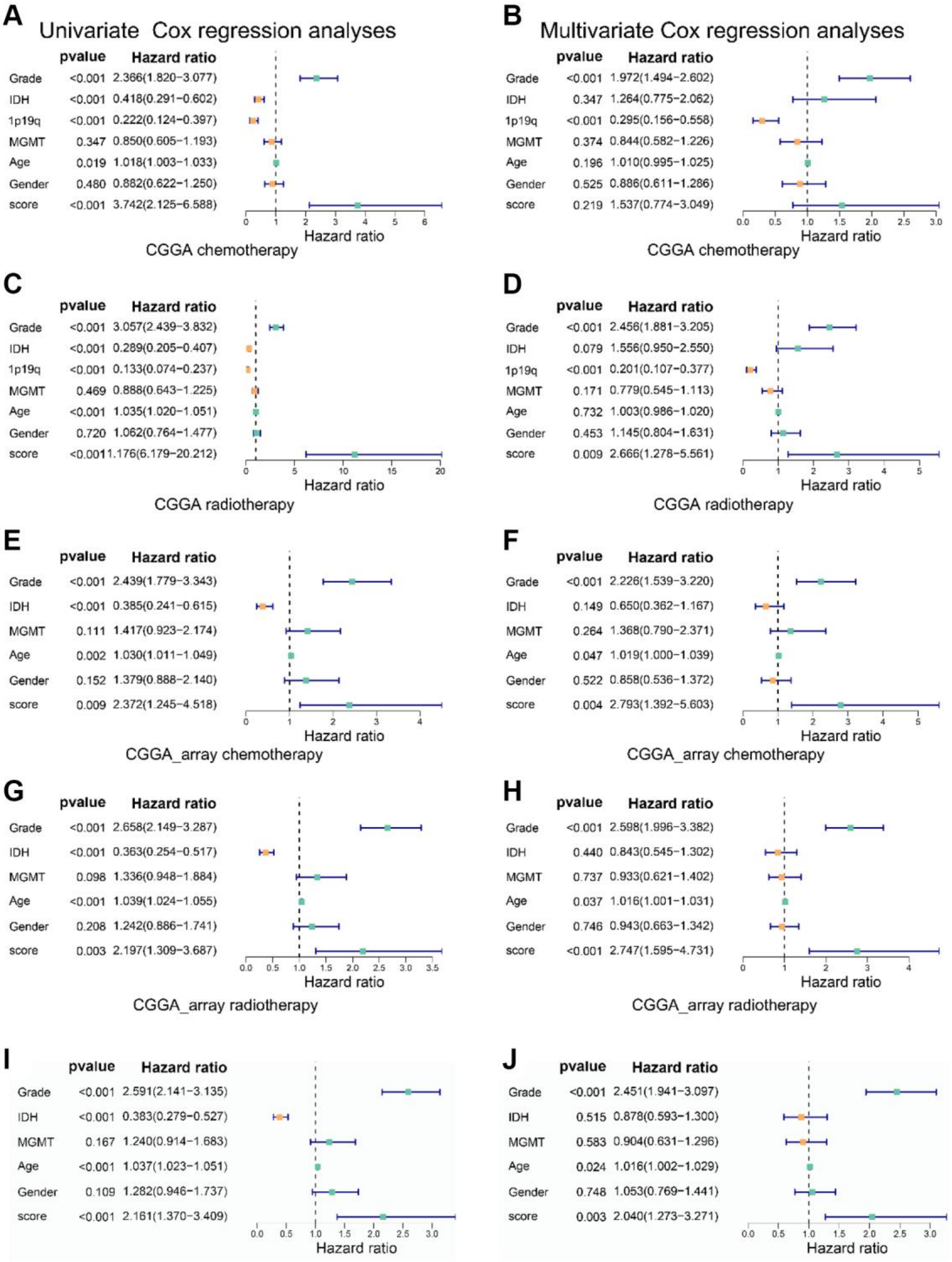

Supplementary Figure 7. COX regression analysis under different conditions. (**A**–**H**) Univariate and multivariate Cox regression analysis of glioma patients receiving different treatment strategies. Univariate (**I**) and multivariate (**J**) Cox regression analysis revealed that angiogenesis score was an independent prognostic factor for glioma patients of CGGA_array dataset.

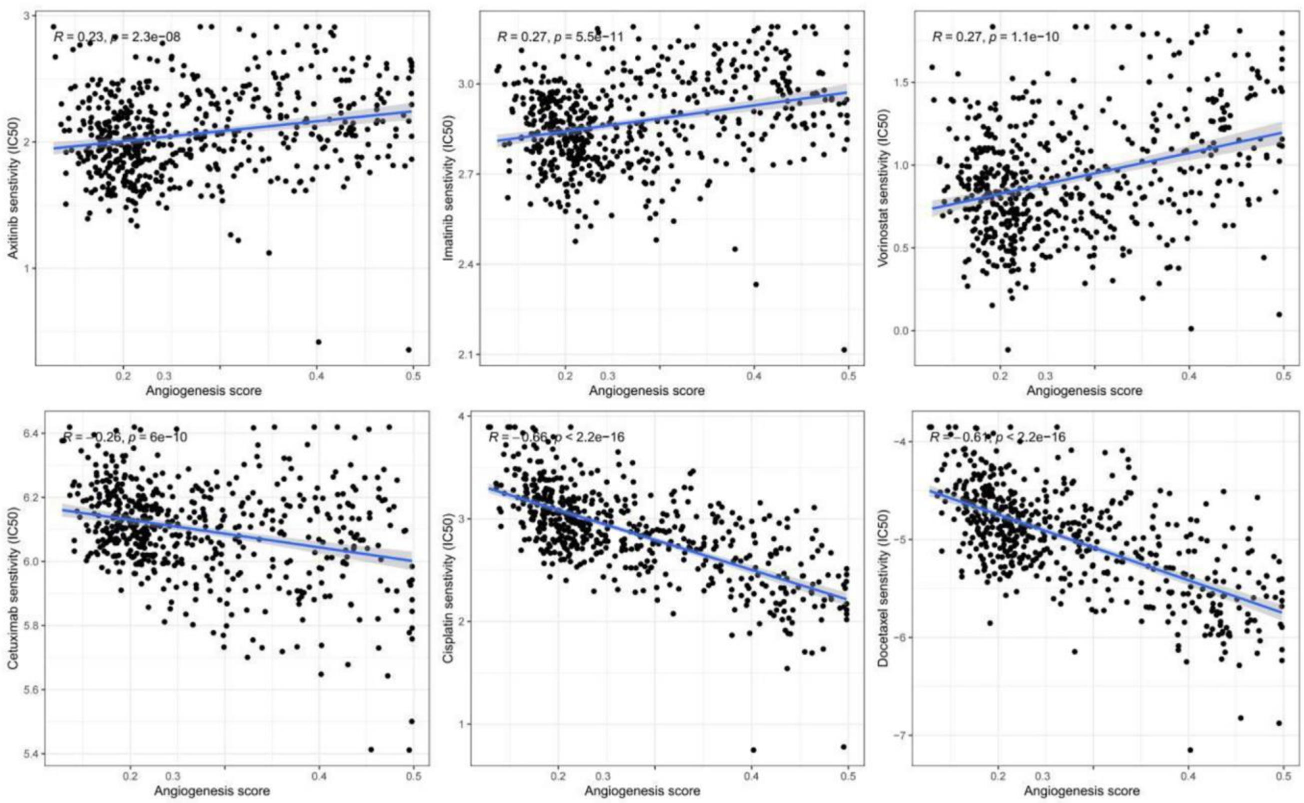

Supplementary Figure 8. Relationships between angiogenesis score and chemotherapeutic sensitivity.
