# Supplementary material for: Intra-tumoral angiogenesis correlates with immune features and prognosis in glioma
Source: Aging (Albany NY). 2022 May 17;14(10):4402–24. doi: 10.18632/aging.204079 (PMC9186765; doi:10.18632/aging.204079)
Supplement: Supplementary Table 1 [file aging-14-204079-s002.pdf]

## SUPPLEMENTARY TABLE

**Supplementary Table 1. Clinical and molecular characteristics of patients included in this study.**

| Cohort | TCGA (*n* = 616) | CGGA (mRNAseq_325) (*n* = 309) | CGGA (array) (*n* = 282) | GSE16011 (*n* = 264) |
|---|---|---|---|---|
| Database | TCGA | CGGA | CGGA | GEO |
| Platform | Illumina Hiseq 2000 RNAseq | Illumina Hiseq 2000 RNAseq | AgilentWholeHuman Genome (Array) | Affymetrix GeneChip Human Genome U133 Plus 2.0 Array |
| Age(year) | | | | |
| Mean (range) | 47.4 (14–89) | 43.2 (8–79) | 42.5 (12–70) | 50.87 (14.38–81.18) |
| Gender | | | | |
| Female | 259 | 116 | 117 | 87 |
| Male | 357 | 193 | 165 | 177 |
| WHO grade | | | | |
| II | 224 | 98 | 106 | 24 |
| III | 243 | 74 | 53 | 85 |
| IV | 149 | 137 | 123 | 155 |
| TCGA subtype | | | | |
| Classical | 83 | – | 23 | – |
| Mesenchymal | 89 | – | 105 | – |
| Neural | 99 | – | 74 | – |
| Proneural | 218 | – | 80 | – |
| Unavailable | 127 | 309 | 0 | 264 |
| IDH status | | | | |
| Mutant | 391 | 166 | 127 | 80 |
| Wild-type | 220 | 142 | 154 | 132 |
| Unavailable | 5 | 1 | 1 | 52 |
| 1p/19q status | | | | |
| Codel | 156 | 62 | 16 | – |
| Non-codel | 455 | 242 | 73 | – |
| Unavailable | 5 | 5 | 193 | 264 |
| MGMT promoter methylation status | | | | |
| Methylated | 441 | 150 | 95 | – |
| Unmethylated | 144 | 141 | 177 | – |
| Unavailable | 31 | 18 | 10 | 264 |

Abbreviations: CGGA: Chinese Glioma Genome Atlas; TCGA: The Cancer Genome Atlas; WHO: World Health Organization; 1p19q Codel: 1p19q codeleted; 1p19qNon-codel: 1p19q non-codeleted.
